# Impaired immune function in Gulf War Illness

**DOI:** 10.1186/1755-8794-2-12

**Published:** 2009-03-05

**Authors:** Toni Whistler, Mary Ann Fletcher, William Lonergan, Xiao-R Zeng, Jin-Mann Lin, Arthur LaPerriere, Suzanne D Vernon, Nancy G Klimas

**Affiliations:** 1 Chronic Viral Diseases Branch, Centers for Disease Control & Prevention, Atlanta, GA, USA; 2 Medical Service, Miami Veterans Affairs Medical Center, Miami, FL, USA; 3 Department of Medicine, University of Miami Miller School of Medicine, Miami, FL, USA; 4 Current address : The Chronic Fatigue and Immune Dysfunction Syndromes (CFIDS) Association of America, Charlotte, NC, USA

## Abstract

**Background:**

Gulf War Illness (GWI) remains a serious health consequence for at least 11,000 veterans of the first Gulf War in the early 1990s. Our understanding of the health consequences that resulted remains inadequate, and this is of great concern with another deployment to the same theater of operations occurring now. Chronic immune cell dysfunction and activation have been demonstrated in patients with GWI, although the literature is not uniform. We exposed GWI patients and matched controls to an exercise challenge to explore differences in immune cell function measured by classic immune assays and gene expression profiling.

**Methods:**

This pilot study enrolled 9 GWI cases identified from the Department of Veterans Affairs GWI registry, and 11 sedentary control veterans who had not been deployed to the Persian Gulf and were matched to cases by sex, body mass index (BMI) and age. We measured peripheral blood cell numbers, NK cytotoxicity, cytokines and expression levels of 20,000 genes immediately before, immediately after and 4 hours following a standard bicycle ergometer exercise challenge.

**Results:**

A repeated-measures analysis of variance revealed statistically significant differences for three NK cell subsets and NK cytotoxicity between cases and controls (p < 0.05). Linear regression analysis correlating NK cell numbers to the gene expression profiles showed high correlation of genes associated with NK cell function, serving as a biologic validation of both the *in vitro *assays and the microarray platform. Intracellular perforin levels in NK and CD8 T-cells trended lower and showed a flatter profile in GWI cases than controls, as did the expression levels of the perforin gene PRF1. Genes distinguishing cases from controls were associated with the glucocorticoid signaling pathway.

**Conclusion:**

GWI patients demonstrated impaired immune function as demonstrated by decreased NK cytotoxicity and altered gene expression associated with NK cell function. Pro-inflammatory cytokines, T-cell ratios, and dysregulated mediators of the stress response (including salivary cortisol) were also altered in GWI cases compared to control subjects. An interesting and potentially important observation was that the exercise challenge augments these differences, with the most significant effects observed immediately after the stressor, possibly implicating some block in the NK and CD8 T-cells ability to respond to "stress-mediated activation". This has positive implications for the development of laboratory diagnostic tests for this syndrome and provides a paradigm for exploration of the immuno-physiological mechanisms that are operating in GWI, and similar complex syndromes. Our results do not necessarily elucidate the cause of GWI, but they do reveal a role for immune cell dysfunction in sustaining illness.

## Background

Some veterans returning from the first Persian Gulf War, Operations Desert Shield and Desert Storm (1990–1991), reported a variety of symptoms including fatigue, musculoskeletal discomfort, skin rashes, and cognitive dysfunction [1-3]. Because many of these veterans potentially experienced various hazards such as physical and psychological stressors, multiple vaccinations, prophylactic medications, infectious agents, pesticides, depleted uranium, oil well fires and smoke, and chemical and biological warfare agents, many hypotheses as to the cause of the syndrome, now called Gulf War Illness (GWI), have ensued [4]. As yet there are no diagnostic clinical signs or laboratory abnormalities that distinguish GWI and the pathophysiology remains inchoate. Thus, there is no specific pharmacological treatment and many of the veterans affected continue to be unwell some 18 years after their return from combat. Perhaps more important, the current deployment of larger numbers of military personnel back to this region, for considerably longer tours of duty, will likely cause considerable morbidity through GWI and similar poorly explained illnesses. Now it is even more important that additional studies are pursued to further our understanding of the illness in order that better treatments are developed. To date, the effects of the numerous different exposures on the veterans are still unclear, but it is likely that many would result in immune function alterations. These have been demonstrated in GWI by several groups [5-7], although the results have not been uniform. We hypothesize that there is a possible heterogeneity to GWI similar to that seen in Chronic Fatigue Syndrome (CFS), and the functional impairment oscillates over the many years of the illness. This makes it difficult to identify the biochemical and physiological measures that are disturbed, as it changes with the symptoms experienced. Along with this, GWI veterans exhibit a post-exertional fatigue that exacerbates clinical symptoms such as pain and cognitive impairment. In an attempt to measure the changing functionality within subjects, we used an exercise challenge paradigm. We believe this would amplify the immune cell dysfunction, allowing us to monitor possible differences in physiologic responsiveness between Gulf war veterans with and without multi-symptom illness. The biological responsiveness was measured using both conventional immunological assays as in the previously published literature, and whole genome expression profiling, in an attempt to understand the pathophysiology of the illness in terms of the complex biological networks.

## Methods

This study was reviewed and approved by Miami University, Department of Veterans Affairs, and Centers for Disease Control and Prevention Institutional Review Boards. All participants were volunteers who gave informed consent.

In this paper we report on a total of 20 subjects (9 GWI and 11 control subjects) taken from a larger study, in an attempt to determine the effectiveness of our approach in evaluating the significant biological differences between GWI cases and controls. Demographic data is summarized in Table 1.

**Table 1:** Demographic and physiological parameters of subjects involved in the exercise challenge paradigm.

	Demographic data	Controls	GWI
Number of subjects		11	9
Race	African American	4	2
	Hispanic	4	6
	White	3	1
Mean Age (years) (Range)		43.8 (33 – 52)	42.8 (34 – 51)
Mean BMI (kg/m^2^) (Range)		26.7 (18.8 – 31.0)	29.1 (24.2 – 31.5)
Mean Peak VO_2 max_^1 ^(ml/kg/min) (± SE)		28.05 (± 1.59)	25.05 (± 0.72)
Mean % Predicted (ml/kg/min) (± SE)		78.36 (± 3.41)*	67.8 (± 1.75)*

* Statistical difference measured by a Student t-Test with equal variance p = 0.015

^1 ^Peak VO_2 max _is the increase in "maximal" oxygen uptake measured during an incremental maximal exercise protocol.

### Subjects

The GWI cases, between the ages of 30 and 55 years old, were identified from the Department of Veterans Affairs' Gulf War Registry, who had no current medical or psychiatric conditions that could preclude GWI as the primary explanation for their fatiguing illness. We also excluded individuals taking medications that could impact immune function (e.g. steroids, immunosuppressive drugs). All cases were veterans deployed to the theater of operations between August 8, 1990 and July 31, 1991 who met the 1998 GWI illness criteria utilized by CDC and the Department of Veterans Affairs [2]. This includes at least one chronic symptom (present longer than 6 months) of fatigue or mood or cognitive complaints and chronic joint pain or stiffness or muscle pain.

As a comparison group we enrolled 11 well veteran controls from the local National Guard units, who defined themselves as sedentary and were of the same age, sex and BMI as the GWI cases (Table 1).

### Exercise challenge

The exercise challenge was performed in the morning, to avoid inter-subject differences in variables measured due to diurnal variation. Subjects arrived at the clinic prior to breakfast, had an intravenous line placement, were served breakfast (contents uniform for all subjects), and lay down for 30 minutes prior to the initial blood draw (T0). Following this, they participated in symptom-limited maximum graded exercise stress test on a bicycle ergometer [8]. This involved pedaling at a power output of 60 watts for 2 minutes and increasing this every 2 minutes, by 30 watts until one of the maximal test criteria were met. These were a plateau in maximal oxygen consumption, a respiratory exchange ratio greater than 1.15 or the subject stopped the test despite vocal urging from the staff All exercise tests were conducted in compliance with the American College of Sports Medicine's published guidelines [9]. We collected a second blood sample (T1) immediately following the challenge (approximately 30 minutes from the start). Subjects then rested for 3 hours following which we obtained a third (T2) blood specimen. There were no adverse events observed as a consequence of the exercise challenge in this study

### Cytokine studies

We measured TNFα, IL10, IL6, IL5, IFNγ, and IL1α using heparanized whole blood collected at each time point. To measure *in vitro *cytokine production we cultured cells for 48 hours at 37°C in 5% CO_2_, either unstimulated or stimulated with phytohemagglutinin (PHA) following which supernatants were collected and frozen at -70°C until analyzed using the Immunotech enzyme-linked immunoassay (ELISA) kits (Beckman Coulter, Hialeah, FL). We also measured *in vivo *plasma TNFα, IL10 and IL6 with a high sensitivity ELISA assay (sensitivity: TNFα – 0.13 pg/ml; IL10 – 0.05 pg/ml; IL6 – 0.02 pg/ml; vendor – Bender MedSystems, Burlingame, CA).

### Salivary cortisol

All subjects submitted 5 saliva samples collected into Salivette tubes (Sarstedt, Germany). Samples were collected at 18:00, the evening prior to the exercise challenge (T-12), at 06:00 (T-2) on the morning of the assessments and exercise challenge, along with blood samples immediately before the morning exercise test (T0), within 5 min of completion of the test (T1) and at 16:00 (T2). Salivary cortisol was measured by a high sensitivity ELISA assay (sensitivity – < .003 μg/dL; vendor – Salimetrics LLC, State College, PA). Assays were run using the Biomek 2000 robotic system with high and low concentrations of cortisol control samples being included in each assay. For this assay, the morning range for healthy adults was 0.940 – 1.551 μg/dL, and the afternoon range, undetectable to 0.359 μg/dl.

### Cell surface phenotyping by flow cytometry

Ethylenediamine tetra-acetic acid anti-coagulated whole blood was surface-stained with optimal dilutions for CD19, CD3, CD4, CD8, CD56, CD16, and CD11a, with isotype controls in four color combinations for 15 min at 25°C. Samples were then fixed and lysed with Optilyse-C reagent, followed by analysis on a FC500 flow cytometer. All reagents and instrumentation were from the Beckman Coulter Corporation (Hialeah, FL). The accuracy and precision of analyses were optimized through the adherence to the CDC's recommendations for flow cytometric analyses [10].

### Flow cytometric assessment of intracellular perforin

The flow cytometric method used for the semi-quantitative assessment of intracellular perforin is published [11].

### NK cell activity

The cytolytic activity of the whole blood was measured against a tumor cell target (K562 cells labeled with ^51^Cr), and relates the number of cells in the sample that are phenotypically NK cells. The assay was performed as described by Maher *et al*. [12].

### Gene expression profiling

Blood was collected into an 8 ml cell preparation tube ((CPT); Becton, Dickinson and Company, Franklin Lakes, NJ) containing sodium citrate at T0 (baseline), T1 (immediately after stressor) and T2 (3 hour recovery). The CPT was processed at the collection site according to the manufacturer's instructions and peripheral blood mononuclear cells (PBMCs) cryopreserved at 5 × 10^6 ^cells/ml freeze medium. Prior to shipping of specimens to the CDC, a 2 ml aliquot of each sample was spun down and the cell pellet resuspended in 1 ml TRIzol reagent. At the CDC laboratories RNA was extracted according to manufacturer's instructions and the RNA quality and quantity assessed using the Agilent 2100 Bioanalyzer. Two μg of total RNA was labeled using the One-Cycle Target Labeling Assay (Affymetrix, Santa Clara, CA, USA) including amplification and labeling controls from the GeneChip^® ^Eukaryotic Poly-A RNA Control Kit. All methodologies followed those suggested by the manufacturer. The fragmented antisense biotin-labeled cRNA had hybridization buffer, Eukaryotic Hybridization Controls (to confirm the sensitivity of hybridization), and OligoB2 controls (positive controls used to orient and grid the array) were added just prior to hybridization to the Affymetrix Human U133 plus 2.0 chips. Hybridization was at 45°C for 16 hours as described in the Affymetrix Users manual [13], followed by washing and staining of arrays with the phycoerythrin-strepavidin conjugate performed using the GeneChip Fluidics Station with the EukGE-WS2v5_450 protocol. Chips were scanned using the Affymetrix GeneChip Scanner 3000 and the Affymetrix GeneChip^® ^Operating Software (GCOS) was utilized for the management, sharing and initial processing of the expression data. Array quality control was performed using the Expression Console™ software (version 1.1, Affymetrix).

The array data files have been deposited in ArrayExpress . Accession number: E-MEXP-2069.

### Data analysis

#### Statistical analysis of immune screening data

To investigate the relationship between immune markers and the case/control groups, multivariate analysis of data from T0 to T1 and T2 time points was performed using repeated-measures analysis of variance ((ANOVA), general linear models procedure). For all the statistical tests applied, 2-tailed p < 0.05 was considered to be statistically significant. SAS statistical software (version 9.1; SAS Institute) was used to conduct the analyses.

#### Gene expression data analysis

The CEL files for each array were imported into BRB ArrayTools (v3.6.0), developed by Dr. Richard Simon and Amy Peng Lam at the National Cancer Institute and Emmes Corporation . Data was normalized using the robust multichip average (RMA) algorithm and a logarithmic (base 2) transformation was applied to the signal intensities. Probe sets showing minimal variation across the arrays were excluded from the analysis, those whose expression differed by at least 1.5 fold from the median in at least 20% of the arrays were retained, giving 9,140 probe sets for further analysis. The Quantitative Trait Analysis (QTA) tool was used to identify genes whose expression was significantly related to the cell numbers of the different lymphocyte subsets. We computed a statistical significance level for each gene testing the hypothesis that the Spearman's correlation between gene expression and each variable was zero. These p- values were then used in a multivariate permutation test [14,15] in which the cell numbers were randomly permuted among the arrays. We used the multivariate permutation test to provide 90% confidence that the false discovery rate (FDR) was < 10%. The FDR is the proportion of false positives in the list of genes claimed to be differentially expressed. The multivariate permutation test is non-parametric and does not require the assumption of Gaussian distributions.

Biological interpretation of the results was achieved using the DAVID Knowledgebase [16,17] for functional annotation and enrichment analysis. The threshold of the EASE score was set at 0.1 (a modified Fisher Exact p-value, where perfect enrichment = 0), with a minimum requirement of 5 genes present in the group. Ingenuity Pathway Analysis (IPA, Ingenuity^® ^Systems; Mountain View, CA. ) was used for network analysis of gene lists.

### Quantitative real-time polymerase chain reaction (qPCR)

Validation of the microarray gene expression findings was performed on all subjects with enough RNA at each time point (3 controls and 3 GWI cases) using qPCR. Eleven genes were validated that were of particular physiological significance, either differentiating GWI cases from controls at T1 (hierarchical clustering data) or that showed up- and down-regulation between time points. The genes are enumerated in additional file 1. Endogenous control genes are required to account for the amount of input RNA, and they need to be equally expressed across illness class and time points. Ten genes were chosen on the basis of the microarray gene expression data that showed low coefficients of variation across all arrays, and also had different biological functions to avoid co-regulated genes. GeNorm [18] was used to select the most stable pair-wise combination of reference genes, which were *PGK1 *and *GAPDH*.

Information on all primers and probe sets used in this validation are given in additional file 1[19]. For each set PCR amplification efficiency (E = 10^-1/slope^) was determined using a 5-step 5 fold dilution standard curve (25 ng to 4 pg) and pooled PBMC total RNA from several donors. One microgram of RNA was reverse transcribed into cDNA using random hexamers for the template cDNA. qPCR was performed using the LightCycler^® ^480 system (Roche Applied Science, Indianapolis, IN) in 96 well plates with Taqman universal PCR master mix (Applied Biosystems, Foster City, CA) according to the manufacturer's instructions using primers and probes outlined in additional file 1. All samples, including the external standards and non-template control were run in triplicate. The 25 μL reaction volume contained 1× Taqman PCR Master Mix, 0.8 μmol/L of each primer, and 2 μL template. The cycling conditions consisted of one cycle at 95°C for 10 min followed by 40 cycles of 95°C × 15 s, 60°C × 15 s and 72°C × 45 s. Data was analyzed using the Lightcycler^® ^480 software (version 1.50).

Initial optimization experiments of the primer-probe sets showed that all PCR products were single bands by agarose gel electrophoresis and that the calibration curves generated from PBMC total RNA (plotting relative concentrations against the threshold cycle (Ct)) had RSq values (an indicator of line fit) from 0.995 to 1.000 for all primer pairs. Based on the slopes of the standard curves, the amplification efficiencies ranged from 1.90 to 2.10.

## Results

GWI cases and control subjects performed similarly during the exercise challenge as shown by comparing their VO_2 max _(ml/kg/min) values, which assesses their level of physical fitness, using a Student t Test. No statistically significant differences were found (Table 1). Therefore, the biological differences that were measured in this study were not related to exertional differences.

### Statistical analysis of immune cell numbers and functional assay data

The changes in immune measures by illness class that showed significant differences (p < 0.05) were NK cytotoxicity (at T0 and T1), CD3- CD56+, CD3- CD16+, and CD3- CD16+ CD11a+ NK cell numbers (all at T2), as well as the response to PHA-stimulation measured in the supernatants of cultured lymphocytes for IL5 and IFNγ (all times) (additional file 2). A significant interaction of time and illness class was observed in CD3- CD56+ cell counts, CD4/CD8 T-cell ratio, PHA-stimulation measures for the Th2 cytokine IL5 and the proinflammatory cytokine TNFα (Additional file 2). Significant time effects (p < 0.001) were observed for all immune variables except NK cytotoxicity, IL6 and IL10 plasma levels, and the *in vitro *PHA cytokine stimulation measures.

The changes in cell subset numbers across time were not as large in the GWI cases compared to controls (not statistically significant (additional file 2)); however, for the CD4/CD8 T-cell ratio, statistically significant differences in illness class, time and the illness class × time interaction were identified (additional file 2). Control subjects had higher CD4/CD8 T-cell ratios at all time points compared to cases, with decreases at T1 for both. This results from an increase in the number of CD3+CD8+ lymphocytes brought into circulation immediately after the stressor (108% from baseline for controls) compared to CD3+ CD4+ (30%). This effect is less pronounced in GWI cases, which show similar CD8 and CD4 T cell numbers to controls at baseline with changes of 64% (p = 0.029) and 25% (p = 0.234) respectively, after the stressor.

### Correlation analysis of gene expression data and immune cell numbers by QTA

The expression of 141 unique probe sets were identified as being correlated to NK cell subset numbers (Spearman's rank correlation coefficients > 0.43), with over 65% of probe sets being common to all 3 subsets. The association between these probe sets and NK cell function was supported by literature searches; of the 141 probe sets identified by QTA, 108 (77%) were strongly implicated in NK cell function (additional file 3).

Examining the gene expression levels of the correlated probe sets between T0-T1 for the control subjects, we found 49 showed a 2-fold increase (additional file 4), and none were decreased using the same criteria. Functional annotation of these genes indicated signal transduction as a predominating biological process, containing 19/49 (39%) of submitted probe sets. Fifteen genes were identified as receptors: *CD247, EDG8, GPR56, IL2RB, KIR3DL2, KIR3DL1, KIR2DL5A, KIR2DS5, KIR2DL2, KLRD1, KLRF1, PTGDR, TRA*, and *TRD*. Examining the expression levels of the GWI subjects only 1 probe set (PTGDR) showed a 2-fold increase in expression, and no probe sets showed a decrease. Repeated measures ANOVA showed that 132 probe sets were statistically significantly different with respect to time, and 82 by illness class, with 74 common to both (additional file 4). No probe sets were significant in the interaction. Closer inspection showed some genes, for example *GFOD1, ZBTB16 *and *RNF165*, were statistically significantly different in both the time and illness class analysis (indicating different expression levels), but showed comparable responses between time points (similar fold changes). Other genes showed differences in expression levels and were less responsive (smaller fold changes) in the GWI cases compared to controls, such as *GZMB, CCL4 *and *FCG3A *(alias *CD16*) (additional file 4). In contrast probe sets identified in the QTA with B-cell numbers showed no statistical significant differences in the gene expression levels by illness class or time.

**Figure 1:**
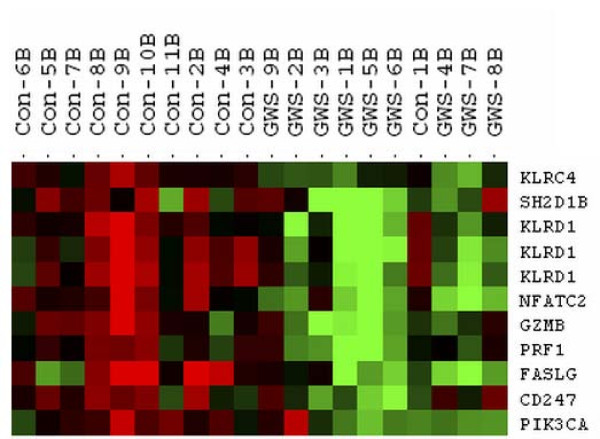
**Heatmap depicting the expression levels of genes separating GWI cases from controls immediately after the exercise challenge**. Immediately after the exercise stress challenge is when we expected differences between GWI cases and controls to be maximal so the most likely time to find the most striking differences. Hierarchical clustering of the 141 probe sets correlating with NK cell subset numbers at the T1 time point was used to show that 9 genes were effective in separating cases from controls. High and low expression levels are shown as red and green respectively. Dendogram at the top shows the clustering results and gene names are given on the right side.

**Figure 2:**
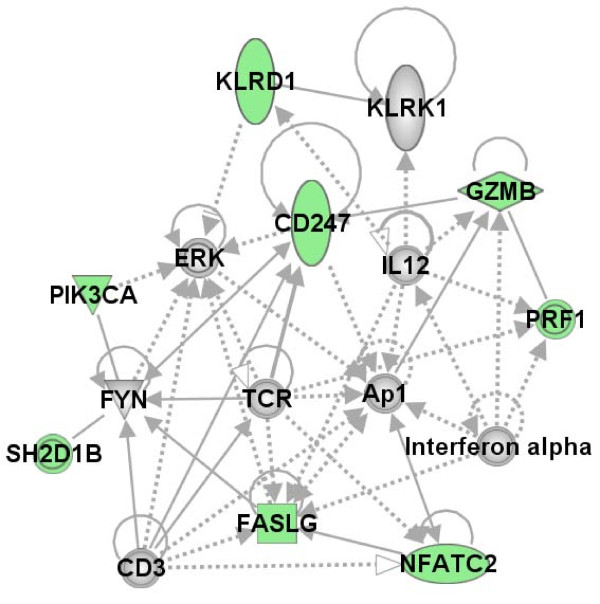
**Functional network of the 9 NK genes differentiating cases from controls**. The genes were overlaid onto a global molecular network developed from information contained in the Ingenuity Pathways Knowledge Base and networks were algorithmically generated based on connectivity. The genes added to the network as connecting molecules are colored grey. The node shape denotes transmembrane receptor (vertical oval), transcription factor (horizontal oval), cytokine (square), kinase (triangle), peptidase (diamond), and a group or complex (double ringed circle). The edges stand for the gene relationship; solid lines indicate a direct interaction, a dashed line an indirect interaction. A solid arrow head between two nodes denotes gene A at arrow base "acts on" gene B at arrow head. Green node color indicates protein correlated to NK cell subset by QTA that differentiates GWI cases from controls.

The genes that differentiated cases from controls in the NK profiles were identified by hierarchical clustering of the 141 unique probe sets (average linkage and centered correlation) using the time point immediately after the exercise challenge (T1), when we expected differences to be the largest. Figure 1 shows the heatmap of 11 probe sets (9 unique genes) that clearly delineated GWI cases from controls. To explore the biological connections between these genes we used the Ingenuity Pathways Knowledge Base and found 8/9 genes formed a single network (Figure 2).

### Intracellular perforin levels

**Figure 3:**
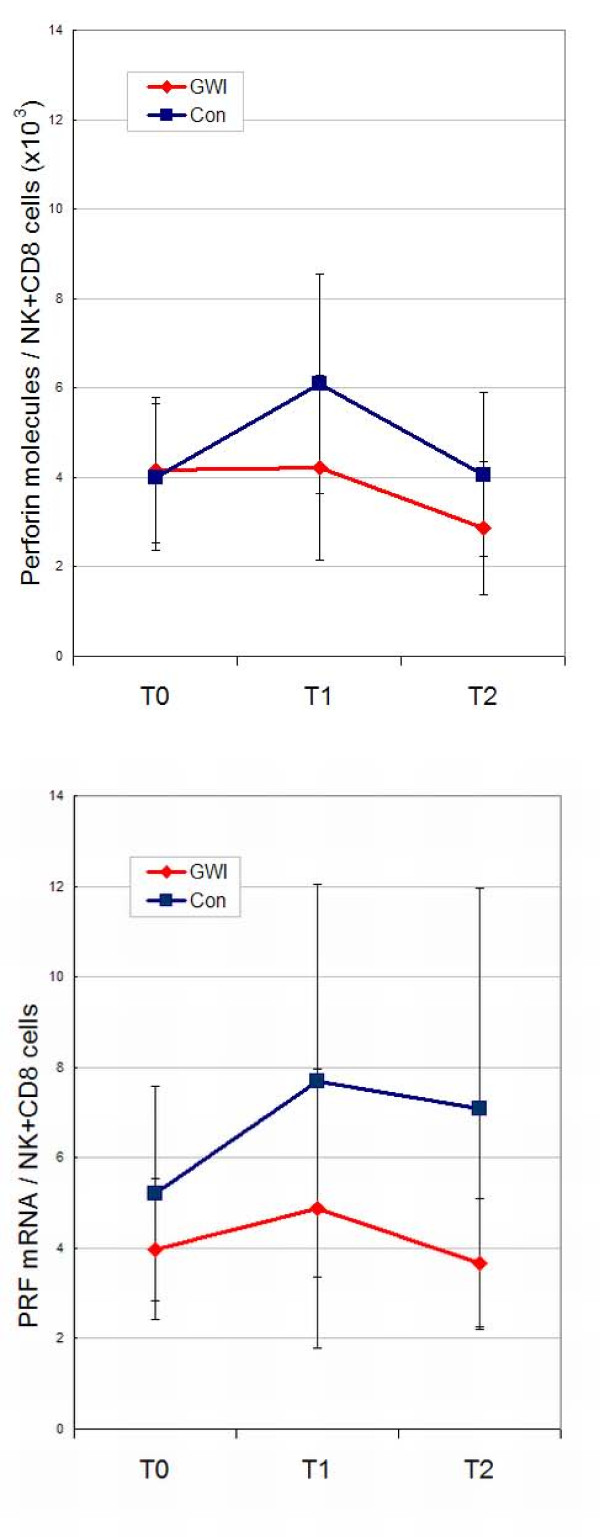
**Change in perforin levels during the exercise challenge time series adjusted for the number of NK and CD8+ cells**. The top graph shows intracellular perforin molecules in both NK and CD8 T-cells and the bottom graph the gene expression data (mean signal intensity). Values are mean ± SD. Repeated measures ANOVA on the intracellular perforin levels showed time series differences bordering on the significant (p-value = 0.053), with illness class differences not significant (p-value = 0.092).

The intracellular perforin levels were measured by quantitative flow cytometry in both NK and CD8+ cells, and the combined data was then corrected to account for the changes in cell numbers during the exercise challenge (Figure 3). The repeated measures ANOVA showed that there were no significant time or illness class effects in the perforin levels (p-values = 0.0539 and 0.0921 respectively), which probably reflects the high variance in the measures and the small number of samples. The gene expression (Figure 3) reflected the protein profiles, with lower mRNA expression level in GWI cases compared to controls.

### Gene expression validation by real-time PCR

The exercise-related changes seen between the T0-T1 and T1-T2 time points were very similar in 9/11 genes used in the validation (additional file 5). Validation of the gene expression data was seen in the lower expression levels for GWI cases compared to controls at all time points in *KLRD1, GZMB*, and *PRF1*, and at T1 for *KLRC4 *(additional file 6). The gene expression patterns of *PIK3CA*, which showed an increase in expression in all 3 time points in controls, and higher expression in GWI cases, was validated by qPCR. (The data for the baseline time point in the GWI cases were not available because of a technical error). Four of 5 genes not part of the hierarchical clustering validated fully (additional file 5). The only discordant result between the gene expression and qPCR data was seen in GWI data for *KLRC4 *and *NCAM1 *between the T2/T1 time points.

## Discussion

The symptom spectrum of GWI is similar to CFS [20], and our results mirror what is seen in the literature with regard to CFS: chronic immune activation [21,22], low cytotoxic immune function [23,24], and dysregulated mediators of the stress response with low baseline salivary cortisol [25] strongly reflecting the overlap between these two syndromes. Decreased functional capacity of NK cells is the one consistent finding in CFS studies [26] and Siegel *et al*. [27] demonstrated that low NK cell function defined a more severely ill cohort. In addition reduced NK cellular cytotoxicity is associated with reduced intracellular perforin [12]. In our study we showed significant group differences in NK cytotoxicity, plus perforin levels that were lower and showed a flatter profile in GWI cases than controls (Figure 3) not reaching statistical significance probably due to small numbers and large variances. Gene expression levels of *perforin *mRNA (*PRF1*) showed similar profiles to the protein levels.

Lymphocytosis induced by exercise is well established in the scientific literature [28,29] and results from the mobilization of immunocompetent cells from immune organs (lymph node and spleen) to the circulation. One to 2 hours into the exercise recovery period the lymphocyte count decreases, sometimes to below pre-exercise levels [30]. Our data confirmed reports that the NK cell subsets are more sensitive to exercise stress than any other cell subtypes [28], but for us it was important to differentiate the causal effects of exercise alone, and to look for the differences between GWI cases and the control subjects. Statistically significant differences were noted for the 3 NK cell subsets between cases and controls, and a significant interaction of illness class and time was observed in the CD3- CD56+ subset. There were no statistically significant differences in the VO_2 max _measures between cases and controls (Table 1) therefore level of exercise performance could not explain these changes. Correlating gene expression to particular cell subsets reflected the cellular shifts in the peripheral blood cells with high statistical significance. For example, 5 NK and T-cell specific surface molecules (*CD160, CD244, CD247, CD38 *and *CD56*) [31] were strongly correlated with the NK subset cell numbers by QTA. To confirm that the gene expression changes between cases and controls evident in our study were not only due to the change in cell numbers, significant expression values were re-evaluated relative to cell numbers. In summary our findings demonstrate control subjects had greater numbers of genes showing expression changes between the T0 and T1 (baseline-stress time points) than GWI cases, and that expression levels in NK cells were consistently lower in GWI cases. However, gene responsiveness varied. Some genes showed a similar responsiveness to the stressor for both GWI cases and controls, whereas others appeared less responsive to the stressor in the cases (additional file 4). The differences in expression levels were greatest at T1 (even when cell number differences were accounted for) but were also present at T0 and T2, when cell numbers were similar. The hierarchical clustering of the NK correlated genes showed that 9 separated GWI cases from controls (Figure 1), each one associated with NK cytotoxicity. All these genes play a role in major cellular functions such as proliferation, activation and apoptosis and the functional network established for 8 of the 9 genes (Figure 2) reveals that 5 of the networked genes (*AP1, CD3, CD247, NFATC2 *and *PIK3CA*) are part of the glucorticoid receptor signaling pathway. Enhanced negative-feedback sensitivity to glucorticoids is often seen in CFS [32,33], as well as a blunted adrenocorticotropin response to stressors [34], and hypocorticolism [35]. This supports the hypothesis that hypo-function of the hypothalamic pituitary adrenal (HPA) axis plays a role in CFS, and probably in GWI also. Disturbances of the HPA axis can be considered as a pathway that links to the immunological disturbances evidenced in CFS and GWI.

Differences in the NK cell receptor mRNAs (*KLRD1, KLRC4*, and *KLRK1*) have been validated between cases and controls (Additional file 2). We know that each NK cell expresses several inhibitory (KIR) and activating (KLR) receptors [36,37], and the balance between the signals triggered by their engagement determines their biological response. The interaction of inhibitory receptors by their cognate ligands (mostly classical and non-classical MHC class I molecules) keeps NK cells inhibited, part of the "missing-self hypothesis" [38]. It has been shown that NK cells that lack inhibitory receptors for self MHC class I molecules are hyporesponsive [39]. Several activating receptors (KLR) have been described, and it is not completely understood how these receptors cooperate with each other, or how they distribute at a clonal level on different NK cells. The effect of down-regulation of receptor mRNA as we see in GWI cases is not known, and how this alters the receptor interactions with cognate ligands is not well understood. This lack of knowledge stems from the heterogeneity and redundancy in the activating receptors which is a drawback to understanding their functional importance in NK cell immunobiology. It will be important to elucidate how these changes affect the cell phenotype and how this moderates the functioning of immune cells. The cytotoxic effector functions of NK cells are mediated by 2 major mechanisms, perforin/granzyme and death receptor induction of apoptosis [40]. In the different analyses performed for this paper, we see that each one of these components is implicated as being different between GWI and control subjects. In fact *PRF1 *and *GZMB*, along with KLR complex receptors and *FASLG *are shown to distinguish cases from controls in the hierarchical clustering, and perforin is implicated in the functional assay measurements (Additional file 2). TNFα differences are noted between GWI cases and controls in the responsiveness of the cultured PBMC to stimulation with PHA. We postulate that the down-regulation of several of the NK receptors in GWI cases could explain the impaired functional response of some lymphocyte subpopulations, as seen by lower gene expression levels in GWI cases of several genes implicated in NK and T-cell biology. It appears that the depressed responsiveness of these genes impacts cell proliferation and activation functions, along with cell signaling and cell survival (apoptosis).

The shift in immune system functioning towards a Th2 (or allergy) profile has been evidenced before in GWI and CFS patients [22,41], as has the contrasting position [6]. Peakman *et al*. [42] reviewed 6 studies that directly addressed the Th2 bias of the immune response as an explanation for GWI and concluded that the data did not support the hypothesis. We found elevated IL-5, a Th2 cytokine, in the GWI cases as compared to controls (Additional file 2); however, the ratio of a Th1 cytokine (IFNγ) to IL-5 was determined and found to show no statistical differences in the repeated measures ANOVA. We found significant differences in CD4/CD8 ratios between cases and controls. The clinical consequences of these changes remain relatively unknown. Data from previous studies showed the ratio was significantly elevated in GWI patients compared to controls [43], the reverse of what we found here. So as with CFS, there are several conflicting reports in the literature regarding immune function [26].

We examined salivary cortisol, a "stress hormone" at baseline (T0), immediately after the exercise stress (T1) and 3 hours later (T2). Control subjects showed increased salivary cortisol from T0 to T1 in our stress paradigm, whereas it decreased in GWI cases. Several conditions are associated with changes in stress system activity [44,45] through modulation of inflammatory responses and the Th1/Th2 balance they may suppress or potentiate disease activity and/or progression. The differences seen here in stress hormones may represent an important mechanism by which stress affects immune-related disease susceptibility, activity, and outcome.

This was a preliminary exploration to determine if an exercise paradigm would help us in understanding the pathophysiology of GWI and if whole genome expression profiling would make a significant contribution to this understanding. For this end we used a small subset of subjects from a much larger study cohort, and two issues arose. Firstly the matching of cases to controls by race fell away (Table 1), and secondly, it was noted that the subjects were brought into clinic in separate groups over a 16 month period, GWI veterans and then control subjects, with little overlap. To address the first issue, we used a multivariate ANOVA with multiple test correction on the gene expression data to find the genes differing by race. None of these were statistically significant in any of the analyses presented in this paper. To manage the latter limitation which would impact class comparison analyses, we focused on differences across the time series. By using within-person paired analysis we could examine exercise-related changes and then compare the results of cases and controls.

## Conclusion

This study shows that exercise induces considerable physiological change in the immune system and the changes observed in GWI cases are less apparent than in control subjects, with differences in the dynamics of the immune response also obvious. Whether the gene expression changes in response to exercise in the circulating immune cells occur because of direct effects in the cells themselves, or are a consequence of mobilizing cells with different expression profiles from various depots is not fully known [46]. It will be important to determine the confounding effects of lymphocyte redistribution. The differences we found are focused in the NK and T-cell populations, involving signal transduction processes possibly through differences in NK receptor expression. We have not elucidated exactly what these changes are, but possible areas of follow-up include the dynamic signaling interactions between NK and T-cells with regard to proliferation, cell cycle differences and activation. The question arises whether the altered number of NK cells is a consequence of the pathological status or a primary condition that leads into the disease.

Another important question is what role do NK cells have in maintaining immune homeostasis in disorders that are thought to involve immune activation such as CFS and GWI? The activities were not correlated with a particular subset of NK cells, and further phenotypic and functional analysis of the different subsets will be necessary to elucidate the immune cells involved in the pathophysiology of GWI. Our data supports the idea of chronic immune cell dysfunction, which appears to be centered on the NK and T-cell lymphocyte populations. There are several plausible explanations for the decrease in NK cell activity. It could result from a shift in NK cell subsets resulting in a larger population of cells with a lower activity profile, or possibly changes in cytokine levels which modulate NK cell activity or the presence of inhibitory substances which could act as ligands binding to the KLR activation receptors.

One of the most interesting and possibly useful observations in this study is that many of the baseline measures show no statistical difference between GWI cases and control subjects. The most significant differences were observed immediately after the exercise challenge, with some of those differences being maintained as far out as 3 hours post-challenge. Thus the exercise challenge paradigm should prove very useful in further elucidation of the disease physiology. This also has positive implications for the development of laboratory diagnostic tests for this and other syndromes such as CFS. The interactions between exercise stress and the immune system as viewed by functional assays and gene expression profiling provide an excellent opportunity to explore the immuno-physiological mechanisms that are operating in GWI, and the possibility of extending this paradigm to other complex syndromes.

## Abbreviations

CCL4: Chemokine (C-C motif) ligand 4; CD: Cluster of differentiation; EDG8: Endothelial differentiation, sphingolipid G-protein-coupled receptor, 8; FASLG: Fas ligand (TNF super family, member 6); FCG3A: Fc fragment of IgG, low affinity IIIa, receptor (CD16a); GAPDH: Glyeraldehyde 3-phosphate dehydrogenase; GFOD1: Glucose-fructose oxido-reductase domain containing 1; GPR56: G protein-coupled receptor 56; IFNγ: Interferon; IL10: Interleukin 10; IL2RB: Interleukin 2 receptor, beta; IL5: Interleukin 5; IL6: Interleukin 6; KIR2DL2: Killer cell immunoglobulin (Ig)-like receptor, two domains, long cytoplasmic tail, 2; KIR2DL5A: Killer cell Ig-like receptor, two domains, long cytoplasmic tail, 5A; KIR2DS5: Killer cell Ig-like receptor, two domains, short cytoplasmic tail, 5; KIR3DL1: Killer cell Ig-like receptor, three domains, long cytoplasmic tail, 1; KIR3DL2: Killer cell Ig-like receptor, three domains, long cytoplasmic tail, 2; KLRC4: Killer cell lectin-like receptor subfamily C, member 4; KLRD1: Killer cell lectin-like receptor subfamily D, member 1; KLRK1: Killer cell lectin-like receptor subfamily K, member 1; MHC: Major histocompatibility complex; MYC: v-myc myelocytomatosis viral oncogene homolog (avian); NCAM1: Neural cell adhesion molecule 1; NFAT2C: Nuclear factor of activated T-cells, isoform2c; PGK1: Phosphoglycerate kinase 1; PIK3CA: Phosphoinositide-3-kinase, catalytic, alpha polypeptide; PRF1: Perforin 1 (pore forming protein); PTGDR: Prostaglandin D2 receptor (DP); RNF165: Ring finger protein 165; TNFα: Tumor necrosis factor (TNF super family, member 2); TRA: T-cell receptor alpha locus; TRD: T-cell receptor delta locus; ZBTB16: Zinc finger and BTB domain containing 16.

## Competing interests

The authors declare that they have no competing interests.

## Authors' contributions

All authors have read and approved the final manuscript.

TW was responsible for oversight of the genomics component of the study, performed the array data analysis and wrote the manuscript.

MAF was the lead study coordinator and oversaw all aspects of the lymphocyte phenotyping, immune function assays and PBMC isolation for the gene expression studies. She participated in designing the study and in the writing of the grant applications that funded the study. She contributed to the writing and editing of the manuscript.

WL performed all aspects of the array workflow and did all initial QC assessment of samples and arrays. WL was also responsible for the qPCR validation. He coordinated specimen handling between sites.

J-MSL performed the analysis on the functional data and advised on the gene expression analysis.

XRZ performed the flow cytometry and functional assays, PBMC collection and shipping, managed the immunology data set, and provided excellent laboratory support and coordination between sites.

AL our exercise physiologist designed and performed the exercise stress testing.

SDV was involved in study design, grant application writing, coordinating interactions between study sites, IRB approval at CDC, and reviewed the manuscript.

NGK was the study PI, who conceived the study design and wrote the VA merit award applications that funded the study. She directed subject recruitment, and was the clinician supervising all medical, stress tests and psychometric evaluations on subjects. She reviewed the manuscript.

## Pre-publication history

The pre-publication history for this paper can be accessed here:



## Supplementary Material

Additional file 1 **Primers and probes sequences used for gene expression validation by real-time PCR**. When available primer/probe sequences were used from the RTPrimerDB [19]. Italicized probes were designed using Primer Express software (v2.0). Where ever possible assays were used that crossed splice junctions. Probes were 5' labeled with 6-carboxyfluorescein (FAM) and 3' labeled with MGB non-fluorescent quencher. Click here for file

Additional file 2 **Statistical analysis of laboratory measures by ANOVA for 2 (illness class) × 3 (time) repeated measures**. All values are given as mean ± standard error of the mean. P-values in **bold **are significant at p < 0.05, those in *italics *are just above this cutoff. A double asterisk indicates significant differences between illness class at indicated time points (**). Significant time effects were determined for all measures (p < 0.001) except those marked with a hash (#). na – measures not taken. * Net concentrations in supernatants of PHA stimulated minus unstimulated blood cultures are expressed as pg/10^5 ^lymphocytes in the culture. * Net concentrations in supernatants of PHA stimulated minus unstimulated blood cultures are expressed as pg/10^5 ^lymphocytes in the culture. Click here for file

Additional file 3 **List of genes that correlated with the NK and B cell subsets in the QTA**. The data provided represent the two gene lists derived from the correlation analysis of the gene expression normalized signal against the natural killer and B-cell numbers respectively. Click here for file

Additional file 4 **Comparison of expression data between GWI cases and controls for the genes correlated with NK cell numbers**. Averaged gene expression data and log ratios of the time series data for GWI cases and controls for the 141 probe sets correlated to NK subset cell numbers. Click here for file

Additional file 5 **Comparison of fold changes in time series data determined by qPCR or gene expression signals for GWI cases and controls**. A graphic representation of these data appears in additional file 6. na – data not available because of technical difficulties. Click here for file

Additional file 6 **Graphical representation of qPCR validation data. Relative quantities of mRNA transcripts in GWI cases and controls as measured by qPCR or oligonucleotide microarray gene expression**. a) Validation results for the differentiation of GWI cases from controls from hierarchical clustering of NK cell number correlation data. b) Validation of the correlation QTA data. Data represents scaled averages of normalized signals ± standard deviation for both real-time RT-PCR data (qPCR) and array expression signal (GE) on samples from GWI cases (hatched bars) and controls (plain bars) for the 3 time points of the exercise challenge: T0 in blue, T1 in red and T2 in yellow. The graph shows similar performance despite different dynamic ranges for the 2 methodologies. For the genes examined expression was lower in cases compared to controls. Click here for file
