# Supplementary figures and images for: Impaired immune function in Gulf War Illness
Source: BMC Med Genomics. 2009 Mar 5;2:12. doi: 10.1186/1755-8794-2-12 (PMC2657162; doi:10.1186/1755-8794-2-12)

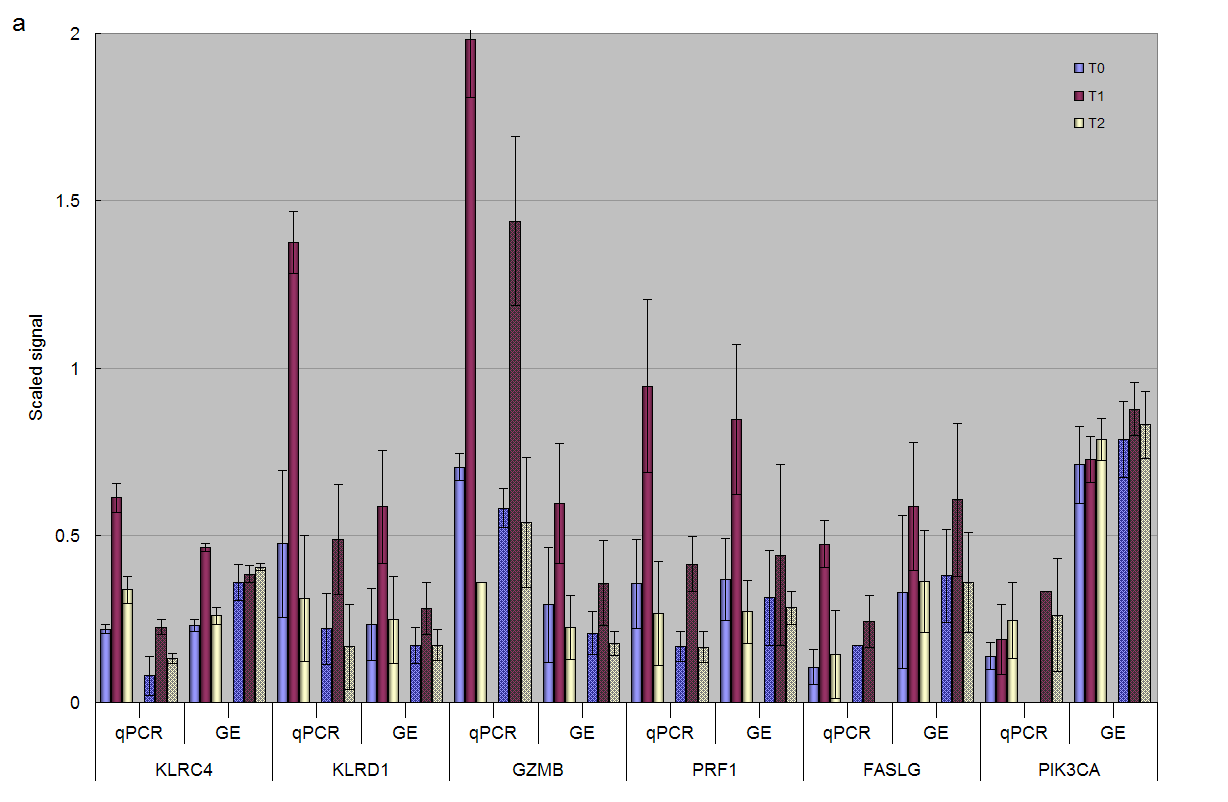


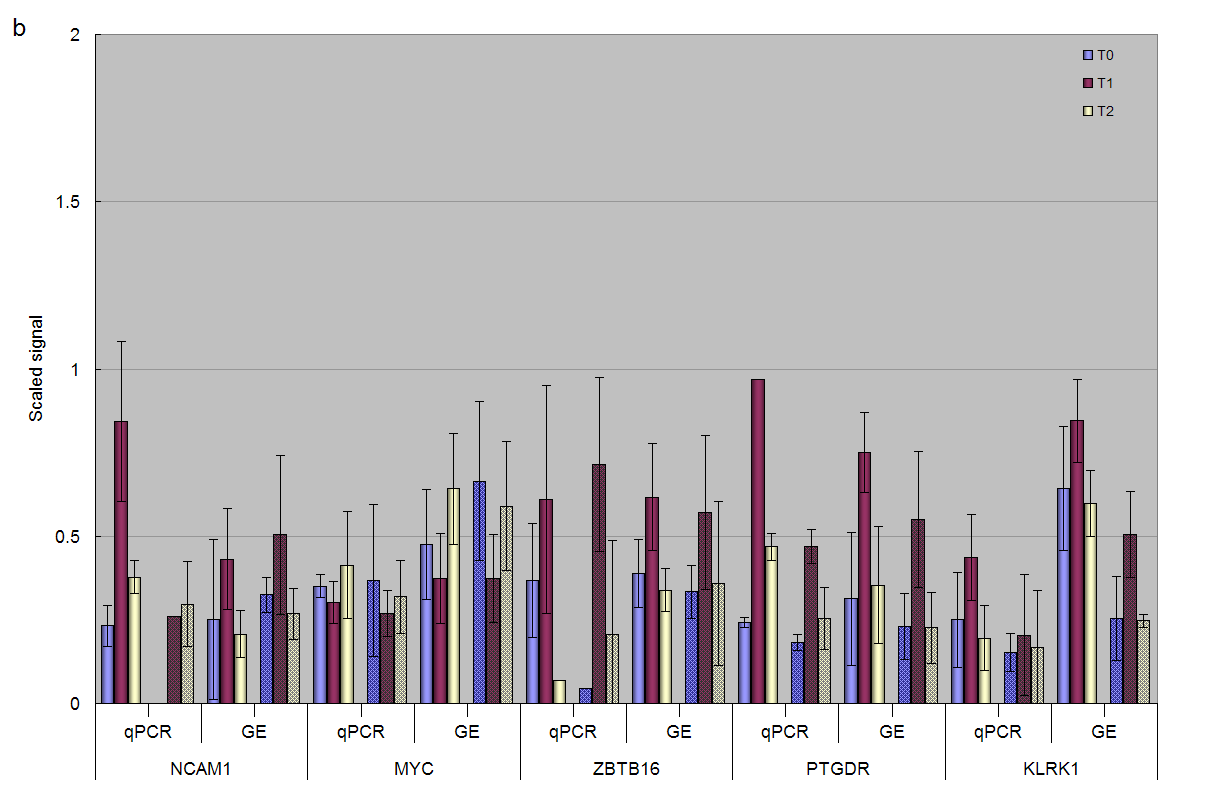

Supplement: Additional file 6 — Graphical representation of qPCR validation data. Relative quantities of mRNA transcripts in GWI cases and controls as measured by qPCR or oligonucleotide microarray gene expression. a) Validation results for the differentiation of GWI cases from controls from hierarchical clustering of NK cell number correlation data. b) Validation of the correlation QTA data. Data represents scaled averages of normalized signals ± standard deviation for both real-time RT-PCR data (qPCR) and array expression signal (GE) on samples from GWI cases (hatched bars) and controls (plain bars) for the 3 time points of the exercise challenge: T0 in blue, T1 in red and T2 in yellow. The graph shows similar performance despite different dynamic ranges for the 2 methodologies. For the genes examined expression was lower in cases compared to controls. [file 1755-8794-2-12-S6.doc]
